# COVID-19 Vaccination Coverage Among Pregnant Women During Pregnancy — Eight Integrated Health Care Organizations, United States, December 14, 2020–May 8, 2021

**DOI:** 10.15585/mmwr.mm7024e2

**Published:** 2021-06-18

**Authors:** Hilda Razzaghi, Mehreen Meghani, Cassandra Pingali, Bradley Crane, Allison Naleway, Eric Weintraub, Tat'Yana A. Kenigsberg, Mark J. Lamias, Stephanie A. Irving, Tia L. Kauffman, Kimberly K. Vesco, Matthew F. Daley, Malini DeSilva, James Donahue, Darios Getahun, Sungching Glenn, Simon J. Hambidge, Lisa Jackson, Heather S. Lipkind, Jennifer Nelson, Ousseny Zerbo, Titilope Oduyebo, James A. Singleton, Suchita A. Patel

**Affiliations:** ^1^CDC COVID-19 Response Team; ^2^Leidos, Inc., Atlanta, Georgia; ^3^Center for Health Research, Kaiser Permanente Northwest, Portland, Oregon; ^4^Division of Healthcare Quality Promotion, National Center for Emerging and Zoonotic Infections, CDC; ^5^Institute for Health Research, Kaiser Permanente Colorado, Denver, Colorado; ^6^HealthPartners Institute, Minneapolis, Minnesota; ^7^Marshfield Clinic Research Institute, Marshfield, Wisconsin; ^8^Department of Research and Evaluation, Kaiser Permanente Southern California, Pasadena, California; ^9^Denver Health, Denver, Colorado; ^10^Kaiser Permanente Washington Health Research Institute, Seattle, Washington; ^11^Vaccine Study Center, Kaiser Permanente of Northern California, Oakland, California.

COVID-19 vaccines are critical for ending the COVID-19 pandemic; however, current data about vaccination coverage and safety in pregnant women are limited. Pregnant women are at increased risk for severe illness and death from COVID-19 compared with nonpregnant women of reproductive age, and are at risk for adverse pregnancy outcomes, such as preterm birth (*1*–*4*). Pregnant women are eligible for and can receive any of the three COVID-19 vaccines available in the United States via Emergency Use Authorization.* Data from Vaccine Safety Datalink (VSD), a collaboration between CDC and multiple integrated health systems, were analyzed to assess receipt of ≥1 dose (first or second dose of the Pfizer-BioNTech or Moderna vaccines or a single dose of the Janssen [Johnson & Johnson] vaccine) of any COVID-19 vaccine during pregnancy, receipt of first dose of a 2-dose COVID-19 vaccine (initiation), or completion of a 1- or 2-dose COVID-19 vaccination series. During December 14, 2020–May 8, 2021, a total of 135,968 pregnant women were identified, 22,197 (16.3%) of whom had received ≥1 dose of a vaccine during pregnancy. Among these 135,968 women, 7,154 (5.3%) had initiated and 15,043 (11.1%) had completed vaccination during pregnancy. Receipt of ≥1 dose of COVID-19 vaccine during pregnancy was highest among women aged 35–49 years (22.7%) and lowest among those aged 18–24 years (5.5%), and higher among non-Hispanic Asian (Asian) (24.7%) and non-Hispanic White (White) women (19.7%) than among Hispanic (11.9%) and non-Hispanic Black (Black) women (6.0%). Vaccination coverage increased among all racial and ethnic groups over the analytic period, likely because of increased eligibility for vaccination^†^ and increased availability of vaccine over time. These findings indicate the need for improved outreach to and engagement with pregnant women, especially those from racial and ethnic minority groups who might be at higher risk for severe health outcomes because of COVID-19 (*4*). In addition, providing accurate and timely information about COVID-19 vaccination to health care providers, pregnant women, and women of reproductive age can improve vaccine confidence and coverage by ensuring optimal shared clinical decision-making.

VSD is a collaboration between CDC’s Immunization Safety Office and nine integrated health care organizations in seven U.S. states; eight sites provide data and one additional site provides subject matter expertise.^§^ Among the eight sites providing data, the integrated health care organizations serve 11.6 million insured persons, including approximately 2.7 million women aged 18–49 years. To monitor vaccination coverage and safety, CDC obtains COVID-19 vaccination data from the VSD sites’ electronic health records, health insurance claims, and state immunization information systems. A dynamic pregnancy algorithm, based on *International Classification of Diseases, Tenth Edition* (ICD-10) diagnosis codes, procedure codes, estimated dates of delivery, and last menstrual period dates from electronic health records was used to identify pregnancies weekly (*5*). Because the algorithm identifies pregnancies based on coded health care utilization data, pregnancies are generally identified at approximately 8–10 weeks’ gestational age. COVID-19 vaccination status was captured for all pregnant women identified from December 14, 2020, when the first COVID-19 vaccine received Emergency Use Authorization, through May 8, 2021. This analysis focused on COVID-19 vaccination during pregnancy. Pregnant women who completed vaccination before pregnancy (1,073) were excluded from this study to ascertain willingness of women to receive the COVID-19 vaccine while pregnant. Receipt of ≥1 dose of a COVID-19 vaccine was defined as receipt of either first or second dose of the Moderna or Pfizer-BioNTech vaccines or receipt of a single dose of the Janssen vaccine during pregnancy. Vaccination initiation was defined as receipt of the first dose of the Moderna or Pfizer-BioNTech vaccines during pregnancy. Vaccination completion was defined as receipt of the second dose (for women who received the first dose before pregnancy) or both doses of Moderna or Pfizer-BioNTech vaccines or 1 dose of Janssen vaccine during pregnancy. COVID-19 vaccination initiation and completion during pregnancy were estimated by age, race and ethnicity, and vaccine type. All analyses were performed using SAS software (version 9.4; SAS Institute). This activity was reviewed by CDC and VSD sites and was conducted consistent with applicable federal law and CDC policy.^¶^

A total of 135,968 pregnant women were identified in VSD during December 14, 2020–May 8, 2021 (Table). Among pregnant women, race and ethnicity data were complete for 93.8% and age data were complete for 100%. White women accounted for 34.0% of pregnancies, and Hispanic women for 32.9%. A larger proportion of pregnant Hispanic women were aged 18–24 years (47.4%) compared with pregnant White (25.4%) and Asian (3.9%) women. Among pregnant women, 16.3% received ≥1 dose of a COVID-19 vaccine; 5.3% initiated, and 11.1% completed vaccination during pregnancy. Vaccination increased with age, with highest rates of ≥1 dose observed among women aged 35–49 years (22.7%) and lowest rates among those aged 18–24 years (5.5%). Receipt of ≥1 dose was highest among Asian women (24.7%), followed by White women (19.7%), and lowest among Black women (6.0%) and Hispanic women (11.9%). The highest rates of receipt of ≥1 dose during pregnancy were reported for Pfizer-BioNTech (8.7%), followed by Moderna (7.0%), and Janssen (0.6%) vaccines. Cumulative receipt of ≥1 dose of a COVID-19 vaccine during pregnancy has increased weekly since March 13, 2021, (when these data were first reported to CDC) among all pregnant women and across all racial and ethnic groups (Figure).

**TABLE:** Receipt of ≥1 dose,* initiation,^†^ and completion^§^ of COVID-19 vaccination during pregnancy among pregnant women, by selected characteristics and by vaccine type — Vaccine Safety Datalink, United States, December 14, 2020─May 8, 2021

Characteristic	No. (%^¶^)								
	Total population	Receipt of ≥1 dose*	Total		Vaccine**				
					Pfizer-BioNTech		Moderna		Janssen
			Initiation^†^	Completion^§^	Initiation^†^	Completion^§^	Initiation^†^	Completion^§^	
**Total**	**135,968 (100)**	**22,197 (16.3)**	**7,154 (5.3)**	**15,043 (11.1)**	**3,658 (2.7)**	**8,226 (6.0)**	**3,496 (2.6)**	**5,992 (4.4)**	**825 (0.6)**
**Age group, yrs**									
18–24	**18,882 (13.9)**	1,044 (5.5)	**458 (2.4)**	**586 (3.1)**	195 (1.0)	307 (1.6)	263 (1.4)	240 (1.3)	39 (0.2)
25–34	**83,335 (61.3)**	13,478 (16.2)	**4,368 (5.2)**	**9,110 (10.9)**	2,203 (2.6)	4,986 (6.0)	2,165 (2.6)	3,638 (4.4)	486 (0.6)
35–49	**33,751 (24.8)**	7,675 (22.7)	**2,328 (6.9)**	**5,347 (15.8)**	1,260 (3.7)	2,933 (8.7)	1,068 (3.2)	2,114 (6.3)	300 (0.9)
**Race and Ethnicity**									
White, NH	**46,245 (34.0)**	9,105 (19.7)	**2,653 (5.7)**	**6,452 (14.0)**	1,456 (3.1)	3,645 (7.9)	1,197 (2.6)	2,491 (5.4)	316 (0.7)
Black, NH	**10,729 (7.9)**	644 (6.0)	**242 (2.3)**	**402 (3.7)**	124 (1.2)	215 (2.0)	118 (1.1)	161 (1.5)	26 (0.2)
Hispanic/Latino	**44,673 (32.9)**	5,312 (11.9)	**1,893 (4.2)**	**3,419 (7.7)**	804 (1.8)	1,689 (3.8)	1,089 (2.4)	1,529 (3.4)	201 (0.4)
Asian, NH	**19,597 (14.4)**	4,834 (24.7)	**1,512 (7.7)**	**3,322 (17.0)**	834 (4.3)	1,880 (9.6)	678 (3.5)	1,252 (6.4)	190 (1.0)
Other, NH^††^	**6,292 (4.6)**	990 (15.7)	**350 (5.6)**	**640 (10.2)**	174 (2.8)	352 (5.6)	176 (2.8)	243 (3.9)	45 (0.7)
Unknown	**8,432 (6.2)**	1,312 (15.6)	**504 (6.0)**	**808 (9.6)**	266 (3.1)	445 (5.3)	238 (2.8)	316 (3.7)	47 (0.6)

**Abbreviation:** NH = non-Hispanic.

* Receipt of first or second dose of the Pfizer-BioNTech or Moderna vaccines or a single dose of the Janssen (Johnson & Johnson) vaccine during pregnancy during December 14, 2020–May 8, 2021.

^†^ Receipt of first dose of Pfizer-BioNTech or Moderna vaccines only during pregnancy during December 14, 2020–May 8, 2021.

^§^ Receipt of both first and second dose, or second dose for women who received the first dose before pregnancy, of Pfizer-BioNTech or Moderna vaccines or receipt of 1 dose of Janssen vaccine during pregnancy during December 14, 2020–May 8, 2021.

^¶^ Percentages might not sum to expected values because of rounding.

** The Food and Drug Administration issued Emergency Use Authorizations for use of COVID-19 vaccines on the following dates: Pfizer-BioNTech, December 11, 2020; Moderna, December 18, 2020; and Janssen, February 27, 2021.

^††^ Includes American Indian or Alaska Native, Native Hawaiian or Pacific Islander, and Multiple or Other races.

**FIGURE:**
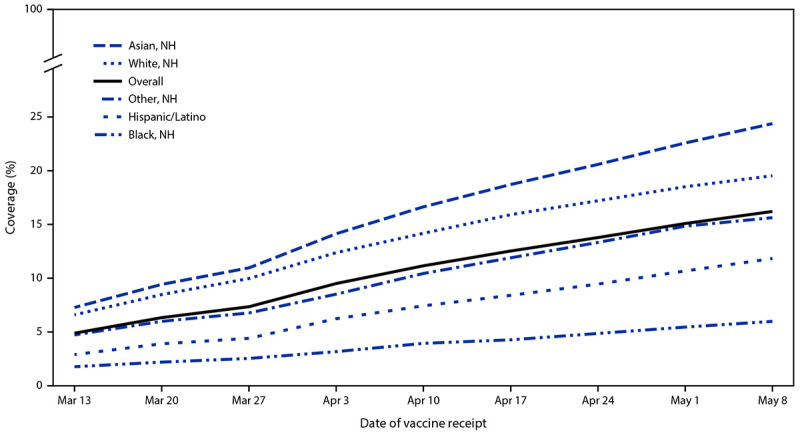
Cumulative COVID-19 vaccination coverage (receipt of ≥1 dose*) among pregnant women,^†^ overall and by race and ethnicity^§^ — Vaccine Safety Datalink, United States, March 13–May 8, 2021^¶^ **Abbreviation:** NH = non-Hispanic. * Receipt of first or second dose of the Pfizer-BioNTech or Moderna vaccines or a single dose of the Janssen (Johnson & Johnson) vaccine. ^†^ All pregnant women identified in the Vaccine Safety Datalink during December 14, 2020–May 8, 2021. These estimates do not exclude pregnant women who completed COVID-19 vaccination before pregnancy. ^§^ “Other, NH” includes American Indian or Alaska Native, Native Hawaiian or Pacific Islander, and Multiple or Other races. ^¶^ Cumulative vaccination data from the Vaccine Safety Datalink were first reported to CDC on March 13, 2021, and included vaccines administered since December 14, 2020; thus, data reported during December 14, 2020–March 12, 2021, could not be displayed by week.

## Discussion

In this analysis, receipt of COVID-19 vaccination during pregnancy was lowest among Black and Hispanic women and women aged 18–24 years; a larger proportion of pregnant Hispanic women were aged 18–24 years compared with pregnant White and Asian women. Similar increasing trends in COVID-19 vaccination coverage have been observed among the general U.S. population as seen among pregnant women (*6*). Even though COVID-19 vaccination coverage has been increasing, Black and Hispanic women still have the lowest vaccination coverage among all racial and ethnic groups. Further, similar results have been reported for receipt of other vaccinations during pregnancy, including influenza and tetanus toxoid, reduced diphtheria toxoid, and acellular pertussis vaccines, in which the lowest vaccination coverage was noted among pregnant Black and Hispanic women** (*7*). These findings highlight racial and ethnic disparities in COVID-19 vaccination coverage to date among pregnant women, who are at increased risk for infection and severe COVID-19–associated illness, indicating a need to prioritize vaccine equity by addressing potential barriers and access issues.

COVID-19 vaccination completion is lower in pregnant women (11.1%) compared with nonpregnant females aged 18–49 years reported in VSD for the same period (24.9%) (CDC, unpublished data, 2021). Low coverage among pregnant women might be attributable to various factors including limited available safety data on COVID-19 vaccines during pregnancy; need for increased vaccine confidence among health care providers and pregnant women; vaccine prioritization, access, and availability; and cultural and language barriers. Coverage differences by vaccine type might be influenced by the date the vaccines were authorized for use, the shorter interval between receipt of first and second doses of Pfizer-BioNTech COVID-19 vaccine than for Moderna vaccine, and vaccine availability at vaccination sites. Pregnant women were excluded from preauthorization clinical trials, and only very limited human data on safety and efficacy during pregnancy were available at the time that the vaccines were authorized for use. Survey data before COVID-19 vaccine authorization showed low acceptance of COVID-19 vaccination among pregnant women, and the most frequently reported reasons for lack of intent to get vaccinated during pregnancy were limited safety data in pregnancy and concerns about possibility of harm to the fetus^††^ (*8*,*9*).

Through early May 2021, COVID-19 vaccination coverage among pregnant women within VSD was low; however, coverage increased over the analytic period across all age and racial and ethnic groups. The increase might be attributable to inclusion of pregnancy among the conditions that increase risk for severe COVID-19 and thus for prioritization for early allocation of COVID-19 vaccines,^§§^ as well as the rollout of vaccines to the entire U.S. population in mid-April. In addition, analyses of emerging data regarding safety of COVID-19 vaccines, specifically mRNA vaccines, have detected no safety signals for pregnant women (*10*). In early data from three of CDC’s vaccine safety monitoring systems, no safety concerns were identified for vaccinated pregnant women or their infants; additional follow-up is needed, particularly among women vaccinated in the first and second trimesters of pregnancy (*10*). There are also emerging data suggesting that COVID-19 vaccination during pregnancy can lead to transfer of antibodies through placenta and breast milk, which might confer some immunity to newborns.^¶¶^

This analysis is the first in the United States to assess COVID-19 vaccination coverage among pregnant women. In addition, this study identifies vaccinations recorded in medical records, health insurance claims, and linked state immunization registries, which minimizes recall or social desirability biases inherent in studies relying on self-reported vaccination. VSD will continue to monitor and assess COVID-19 vaccination among pregnant women weekly.

The findings in this report are subject to at least four limitations. First, the findings might not be generalizable to all pregnant women in the United States because VSD collects data within eight integrated health care organizations. Second, vaccination status could be misclassified in VSD if some pregnant women received vaccinations outside of participating vaccine delivery systems or state registry catchment areas. Third, data on some covariates of interest (especially race and ethnicity) are incomplete in VSD data, although more complete than the national vaccination data reported by CDC (*6*). Finally, the dynamic pregnancy algorithm might result in some misclassification of pregnancy status and dates, especially in weekly reports when data from ongoing pregnancies might be incomplete.

Although low, COVID-19 vaccination coverage among pregnant women is expected to increase as vaccine availability and access improve, and as more safety data become available. Addressing barriers to access as well as augmenting the scientific evidence regarding safety and effectiveness of COVID-19 vaccines in pregnancy are critical. In addition, vaccine misinformation and hesitancy should be addressed. Strategies and approaches to expanding vaccination coverage in ways to ensure and prioritize equity also should be implemented.*** Finally, making accurate and timely information available to health care providers and pregnant women could increase confidence and thus acceptance of COVID-19 vaccines in this population.

**Summary**

**What is already known about this topic?**

Pregnant women are at increased risk for severe illness and death from COVID-19.

**What is added by this report?**

As of May 8, 2021, 16.3% of pregnant women identified in CDC’s Vaccine Safety Datalink had received ≥1 dose of a COVID-19 vaccine during pregnancy in the United States. Vaccination was lowest among Hispanic (11.9%) and non-Hispanic Black women (6.0%) and women aged 18–24 years (5.5%) and highest among non-Hispanic Asian women (24.7%) and women aged 35–49 years (22.7%).

**What are the implications for public health practice?**

Improving outreach to and engagement with health care providers and pregnant women, especially those who are younger and from racial and ethnic minority groups, could increase vaccine confidence and thus coverage of COVID-19 vaccination in this population.

## Footnotes

*
https://www.cdc.gov/vaccines/covid-19/clinical-considerations/covid-19-vaccines-us.html

†
https://www.cdc.gov/mmwr/volumes/69/wr/mm695152e2.htm

§
https://www.cdc.gov/vaccinesafety/ensuringsafety/monitoring/vsd/

¶ 45 C.F.R. part 46.102(l)(2), 21 C.F.R. part 56; 42 U.S.C. Sect. 241(d); 5 U.S.C. Sect. 552a; 44 U.S.C. Sect. 3501 et seq.

**
https://www.cdc.gov/flu/fluvaxview/dashboard/vaccination-coverage-pregnant.html

††
https://www.medrxiv.org/content/10.1101/2021.03.26.21254402v1

§§
https://www.cdc.gov/coronavirus/2019-ncov/need-extra-precautions/people-with-medical-conditions.html

¶¶
https://www.ajog.org/article/S0002-9378(21)00187-3/fulltext

***
https://aspe.hhs.gov/system/files/pdf/265511/vaccination-disparities-brief.pdf

## References

[1] Woodworth KR, Olsen EO, Neelam V, ; CDC COVID-19 Response Pregnancy and Infant Linked Outcomes Team. Birth and infant outcomes following laboratory-confirmed SARS-CoV-2 infection in pregnancy—SET-NET, 16 jurisdictions, March 29–October 14, 2020. MMWR Morb Mortal Wkly Rep 2020;69:1635–40. 10.15585/mmwr.mm6944e2 33151917 PMC7643898

[2] Wei SQ, Bilodeau-Bertrand M, Liu S, Auger N. The impact of COVID-19 on pregnancy outcomes: a systematic review and meta-analysis. CMAJ 2021;193:E540–8. 10.1503/cmaj.202604 33741725 PMC8084555

[3] Allotey J, Stallings E, Bonet M, ; for PregCOV-19 Living Systematic Review Consortium. Clinical manifestations, risk factors, and maternal and perinatal outcomes of coronavirus disease 2019 in pregnancy: living systematic review and meta-analysis. BMJ 2020;370:m3320. 10.1136/bmj.m3320 32873575 PMC7459193

[4] Zambrano LD, Ellington S, Strid P, ; CDC COVID-19 Response Pregnancy and Infant Linked Outcomes Team. Update: characteristics of symptomatic women of reproductive age with laboratory–confirmed SARS-CoV-2 infection by pregnancy status—United States, January 22–October 3, 2020. MMWR Morb Mortal Wkly Rep 2020;69:1641–7. 10.15585/mmwr.mm6944e3 33151921 PMC7643892

[5] Naleway AL, Crance B, Irving SA, Vaccine Safety Datalink infrastructure enhancements for evaluating the safety of maternal vaccination. Ther Adv Drug Saf 2021. Epub June 14, 2021. 10.1177/20420986211021233 PMC8207278 34178302

[6] CDC. CDC data tracker: demographics trends of people receiving COVID-19 vaccination in the United States. Atlanta, GA: US Department of Health and Human Services; CDC; 2021. https://covid.cdc.gov/covid-data-tracker/#vaccination-demographics-trends

[7] Razzaghi H, Kahn KE, Black CL, Influenza and Tdap vaccination coverage among pregnant women—United States, April 2020. MMWR Morb Mortal Wkly Rep 2020;69:1391–7. 10.15585/mmwr.mm6939a2 33001873 PMC7537555

[8] Goncu AS, Oluklu D, Atalay A, COVID-19 vaccine acceptance in pregnant women. Int J Gynaecol Obstet 2021. E-pub April 19, 2021. 10.1002/ijgo.13713 33872386 PMC9087778

[9] Skjefte M, Ngirbabul M, Akeju O, COVID-19 vaccine acceptance among pregnant women and mothers of young children: results of a survey in 16 countries. Eur J Epidemiol 2021;36:197–211. 10.1007/s10654-021-00728-6 33649879 PMC7920402

[10] Shimabukuro TT, Kim SY, Myers TR, Preliminary findings of mRNA Covid-19 vaccine safety in pregnant persons. N Engl J Med 2021. E-pub April 21, 2021. 10.1056/NEJMoa2104983 PMC8117969 33882218

